# Health-related quality of life in young Syrian refugees recently resettled in Norway

**DOI:** 10.1177/1403494820929833

**Published:** 2020-07-02

**Authors:** Cecilie R. Dangmann, Øivind Solberg, Anne K.M. Steffenak, Sevald Høye, Per N. Andersen

**Affiliations:** 1 Inland Norway University of Applied Sciences, Norway; 2 Norwegian Centre for Violence and Traumatic Stress Studies, Norway; 3 Inland Norway University of Applied Sciences and Nord University, Norway

**Keywords:** Refugee, adolescent, health-related quality of life, Syria, KIDSCREEN-27, physical well-being, psychological well-being, parents, school, friends

## Abstract

*Aims:* Millions have fled from the civil unrest in Syria, and half of these are children and youth. Although they are a population with an elevated risk of health problems due to adverse pre-migratory and post-migratory experiences, few studies have explored their health-related quality of life (HRQoL). This is considered a fundamental construct in public health and might provide complementary descriptions of their health and well-being after resettling in a new country. *Methods:* This was a cross-sectional study of 160 Syrian youth aged 13–24 years. Using KIDSCREEN-27, the results for five dimensions of HRQoL was compared to population norm data. Demographic factors and war-related adverse events were used to predict HRQoL in hierarchical regression. *Results:* For most participants, the overall HRQoL was good, but it was lower in the dimensions for friends, physical well-being and psychological well-being compared to population norms. Scores in the dimensions for autonomy/parental relation and the school environment were high and were the main contributors to a positive HRQoL. Age and number of reported stressful events (SE) had the greatest impact on HRQoL, but the final regression model only accounted for 21% of the total variance. ***Conclusions:* HRQoL is a relevant and non-invasive measure for refugee youth. Contributors to lower scores in physical and psychological well-being should be explored further and indicate the potential for future interventions focussing on general psychological well-being and networks, regardless of the SE that have been experienced. These interventions could potentially be based in schools or in families in order to benefit from these being seemingly safe environments for the majority of the group.**

## Introduction

The global population of forcibly displaced people has grown to more than 70 million [1]. The armed conflict in Syria has caused one of the largest mass movements since World War II, with more than six million on the move. Most are internally displaced, but millions also cross the borders to neighbouring countries such as Jordan and Turkey. About one million have fled to Europe and received asylum, with Germany and Sweden receiving the highest number of such applications at 550,000 claims [2]. Syrians are also one of the largest groups of refugees in Norway, totalling about 22,000, and almost all are granted permanent settlement [3]. Half of the displaced Syrians are children <18 years of age [2].

Many Syrian children have experienced war and violence at close hand and have been forced to move, leaving their home, family and friends. They have seen or heard guns, bombs and fighting; many have seen someone die, and sometimes these were family members or loved ones [4–6]. Pre-migratory adverse events and the complexities of post-migration settlement involving language acquisition and sociocultural adjustment are associated with a range of negative symptoms such as anxiety, depression and post-traumatic stress disorder, and this has been the main focus of an expanding literature on mental health in refugee children and youth [7–11]. However, the majority of refugee youth do not develop psychiatric problems, despite their traumatic experiences [12], and the symptoms often reduce over time [13]. Therefore, knowledge of their general health and well-being is important to monitor health well-being in the whole group. The concept of health-related quality of life (HRQoL) might offer complementary insight, allowing a broader focus on health outcomes not limited to risk and vulnerability, in line with recommendations in the fields of both migration and public-health research [14].

HRQoL is a multidimensional construct covering physical, emotional, mental, social and behavioural components related to health as perceived by the individuals themselves [15]. Some of the related determinants are age, sex, socio-economic status and health [16].

Previous studies have indicated slightly lower well-being among immigrant populations. However, results often vary due to the heterogeneity of the concepts measured, migrant groups included and context [7,17]. For refugee youth, as a subgroup of immigrants, the results also vary. Two Australian studies found HRQoL levels comparable to rates in healthy populations [18,19], whilst a Swedish study found lower scores for foreign-born youth [20], and a UN project reported substantially lower HRQoL scores for Syrian children living in Jordan [21]. In addition to age and sex, some of the related determinants were region of origin, residence time, family situation and war-related events [18,20].

Due to limited knowledge of HRQoL in refugee youth and conflicting results in previous studies, the purpose of this study was to explore the HRQoL in young Syrian refugees who have recently resettled in Norway, and its relationship with settlement factors and war- and flight-related events. The results might help discern dimensions of strengths and areas of concern for refugee youth who resettle in high-income countries.

## Methods

The study has a cross-sectional design using a questionnaire in both Arabic and Norwegian. Using strategic sampling to recruit recently resettled youth, 40 schools with introductory classes for newly arrived immigrants were contacted. Of these, 23 schools located in nine different regions of Norway agreed to participate. Reasons for not participating were: no response to request (*n*=9 schools), no Syrian students (*n*=6 schools) or already participating in other studies (*n*=2 schools). The schools participating had between 1 and 23 Syrian students attending. The teachers distributed written information about the study in Arabic and Norwegian to the students in advance, and consent forms were given to parents with children <16 years of age. The youth completed the questionnaire whilst at school with a researcher present to answer questions. Norwegian schools do not regularly record students’ country of origin. Therefore, the total number of Syrian students attending each school was not available. Of the students present on the day of the visit, three students declined to participate due to exam preparations or language difficulties. Students absent from school were not included. Due to challenges collecting consent forms from parents of children aged <16 years, efforts were focussed on including upper secondary schools.

### Measures

#### HRQoL

The KIDSCREEN-27 tool was used to assess subjective HRQoL. This is a generic self-report measure used in both healthy and ill children aged 8–18 years all over the world. It is a reliable, sensitive and cross-culturally validated measure in 38 languages [22]. It measures five HRQoL dimensions: physical well-being (physical), psychological well-being (psychological), autonomy and parent relations (autonomy/parents), social support and peers (friends) and school environment (school). We also included the HRQoL Index, which consists of 10 items from all dimensions.

Previous research has shown acceptable reliability of the scale, with a Cronbach’s alpha >0.80 [23] and acceptable Rasch measurements [16], also for older youth [24]. In this study, Cronbach’s alpha was between 0.82 and 0.88 for all dimensions included, suggesting good internal consistency for the questionnaire. Population norm data for the KIDSCREEN-27 tool based on a large sample of children and youth from 13 different European countries were available for comparison [16]. Higher scores indicate higher self-rated HRQoL.

#### Stressful events

Adverse war- and flight-related experiences have different connotations in the literature. Based on previous research, we modified a list of potentially traumatic or stressful events (SE) to fit the age and context of the participants [25]. The list consisted of 10 dichotomous items (yes/no): witnessing war, forced to leave friends/family, someone you love disappearing, someone trying to hurt you or someone you love, having a life threatening illness or injury, lacking food or shelter, having to hide, torture, seeing someone die and other frightening experience where you thought your life was in danger.

#### Sociodemographic and settlement factors

Age, sex, mother tongue, time as a refugee (time since they left their own house in Syria), residence time (time since arrival in Norway), moves (number of times they have moved during the last five years) and family situation were included. The latter was dichotomised into living with parents (one or both parents, or one parent and step-parent) or not living with parents (living alone, in an orphanage or other).

### Statistical analyses

All KIDSCREEN-27 scores were summed and converted to sum scores with a range of 0–100 according to KIDSCREEN scoring manuals. In dimensions with one missing value, the missing value was replaced with the individual mean for the dimension estimated by the remaining items [16]. The comparison of mean HRQoL sum scores in the study and population norms were tested with an independent two-tailed *t*-test using Bonferroni corrections for multiple comparisons. One-way analysis of variance (ANOVA) was used to investigate differences in mean HRQoL scores between three age groups in the sample. Lastly, Pearson’s correlation coefficients together with hierarchical multiple regression were used to explore the relationships among the variables. All analyses were conducted using IBM SPSS Statistics for Windows v21.0 (IBM Corp., Armonk, NY), with statistical significance set at *p*<0.05 (two-tailed). This study was reviewed and approved by the Regional Research Ethics Committee of Norway East (reference number 2018/192).

## Results

### Characteristics of the sample

In total, 160 youth were included in the study. The mean residence time in Norway for the study population was two years (range 0–8 years). The mean age was 18.6 years (range 13–24 years). The sex distribution was slightly uneven, with 62.5% boys and 36.9% girls. For the majority of the youth (76.3%), Arabic was their mother tongue, and they lived with their parents (75%; see Table I). The youth not living with their parents were mostly >19 years of age (*M*_age_=20.1 years, range 16–24 years), lived alone and were mostly male (89%).

**Table I.** Characteristics of the sample.

Demographic variables	*n* (%), *N*=5553	*n* (%), *N*=160
Age (mean)	18.7 years	18.6 years
13–16 years	1804 (32.5)	48 (30)
17–18 years	836 (15)	47 (29.4)
19–24 years	2913 (52.5)	65 (40.6)
Sex		
Boys	3523 (63.5)	100 (62.5)
Girls	2030 (36.6)	59 (36.9)
Mother tongue		
Arabic		121(76.3)
Kurdish		34 (21.9)
Other		3 (1.8)
Time as refugee		5.3 years
Number of moves		3.7 times
Residence time (mean)		2.0 years
<2 years		62 (38.8)
⩾2 years		85 (53.1)
Living with parents		
Yes		120 (75)
No		35 (21.9)

From data obtained from Statistics Norway (SSB), we know that there were 5553 Syrian youth between the ages of 13 and 24 years registered in Norway at the start of the study. The mean age and sex distribution in this population is close to that of our study population.

### SE

Participants (*n*=147) reported a mean of 4.2 SE (*SD*=2.76), the most prevalent being witnessing war (68%), feeling your life was in danger (59%) and seeing someone die (55%). A total of 88% reported at least one event, with 61% reported having experienced four or more events.

### HRQoL

The mean HRQoL Index for the sample was 64.06 (*SD*=18.97; range from 10–100). The dimensions where the youth indicated best satisfaction were autonomy/parents, with a mean score of 69.50 (*SD*=21.43) and school, with a mean score of 67.25 (*SD*=21.40). The dimensions with the lowest scores were physical and psychological well-being, with mean scores of 58.82 (*SD*=23.00) and 59.02 (*SD*=21.07), respectively. This is a distinctly different HRQoL profile than the European population norm data, where friends and psychological well-being are the dimensions with the highest scores, and school the lowest (see Table II).

**Table II.** Comparison between HRQoL scores in study population and European population norms.

HRQoL dimensions	Study (13–24 years)			European population norms (12–18 years)			*t*	*p*	Cohen’s *d*^a^
	*n*	*M*	*SD*	*n*	*M*	*SD*			
Physical	157	58.82	23.00	15,239	68.24	18.71	6.26	<0.001***	0.5
Psychological	155	59.02	21.07	15,239	75.06	16.95	11.66	<0.001***	0.9
Parents	156	69.50	21.43	15,239	72.98	18.7	2.31	0.02*	0.2
Friends	160	65.64	25.53	15,239	76.29	19.7	6.78	<0.001***	0.5
School	160	67.25	21.40	15,239	66.25	19.9	0.63	NS	
HRQoL Index	157	64.06	18.07	14,932	71.93	15	6.52	<0.001***	0.5

In dimensions missing one value, the missing value was replaced by the mean of the remaining values. Total missing values: Physical: 8 missing one value, 3 missing more than one value. Total: 11. Psychological: 12 missing one value, 5 missing more than one value. Total: 17. Parents: 1 missing one value, 4 missing more than one value. Total: 5. Friends: 1 missing one value, 0 missing more than one value. Total: 1. School: 1 missing one value, 0 missing more than one value. Total: 1.

a Effect size (convention): 0.20=small; 0.50=moderate; 0.80=large.

* *p*⩽0.05; **p⩽0.01; ***p⩽0.001. Significance after Bonferroni correction in shown in bold.

HRQoL: health-related quality of life; NS=not significant.

When we compare each dimension to the European population norms, we find that the HRQoL Index is significantly lower in our sample (64.06 vs. 71.93) with a moderate effect size. The three dimensions that contribute to the lower HRQoL Index are physical well-being, psychological well-being and friends. The effect size for the difference in physical well-being and friends are moderate, but for psychological well-being they are large.

The age range of the European population norm data is up to 18 years of age, whereas we included youth up to 24 years of age. Assuming age was relevant to HRQoL, we ran the same comparison including only our study participants in the same age range as a sensitivity analysis. The adjustment increased the mean scores for all dimensions in our study sample (see Table III). The significant differences between the study sample and the population norms remained, but the effect sizes diminished.

**Table III.** Comparison between HRQoL scores in study population (age 13–18 years) and European population norms.

HRQoL dimensions	Study (13–18 years)			European population norms (12-18 yrs)			*t*	*p*	Cohen’s *d*^a^
	*n*	*M*	*SD*	*n*	*M*	*SD*			
Physical	93	62.75	23.1	15,239	68.24	18.7	2.82	0.005**	0.3
Psychological	93	63.12	21.42	15,239	75.06	17	6.74	<0.001**	0.7
Parents	91	72.93	22.07	15,239	72.98	18.7	0.03	NS	
Friends	94	68.77	24.15	15,239	76.29	19.7	3.68	<0.001**	0.4
School	94	70.53	22.34	15,239	66.25	19.9	2.08	0.038*	0.2
HRQoL Index	92	67.53	18.51	14,932	71.93	15	2.80	0.005**	0.3

a Effect size (convention): 0.20=small; 0.50=moderate; 0.80=large.

* *p*⩽0.05; **p⩽0.01. Significance after Bonferroni correction shown in bold.

In further analysis, we investigated the impact of different sociodemographic variables, including settlement factors, starting with age. Dividing the sample into three age groups, Table IV shows the mean HRQoL values and results of the ANOVA. This shows a gradual decrease in the mean scores for all dimensions of HRQoL with increasing age, with the oldest age group scoring significantly lower than the youngest age group. However, with Bonferroni correction, only psychological well-being and autonomy/parents remained significantly different, suggesting that these dimensions contribute the most to the difference in the HRQoL Index between the youngest and the oldest. However, the effect sizes were relatively small.

**Table IV.** Mean values and results for ANOVA for study sample divided into age groups.

HRQoL dimensions	13–16 years Group 1 (*N*=46)	17–18 years Group 2 (*N*=47)	19–24 years Group 3 (*N*=63)	*F* (*df*)	*p*	Post-hoc groups	η^2^
Physical	63.84 (25.1)	61.03 (21.3)	53.42 (21.9)	3.1 (2, 154)	0.046	1 vs. 3	0.04
Psychological	65.64 (23.4)	60.02 (19.2)	53.15 (19.1)	5.0 (2, 152)	**0.008**	1 vs. 3	0.06
Parents	77.42 (22.3)	68.48 (20.9)	64.53 (19.8)	5.2 (2, 153)	**0.007**	1 vs. 3	0.06
Friends	73.05 (23.6)	63.87 (24.1)	61.44 (27.1)	3.1 (2, 157)	0.048	1 vs. 3	0.04
School	74.22 (23.3)	66.58 (20.7)	62.60 (21.4)	4.3 (2, 157)	0.016	1 vs. 3	0.05
HRQoL Index	70.9 (19.9)	63.8 (16.4)	59.3 (16.5)	5.9 (2, 154)	**0.003**	1 vs. 3	0.07

Significance after Bonferroni correction shown in bold.

Effect size η^2^ (convention): 0.01=small; 0.06=moderate; 0.14=large.

ANOVA: analysis of variance.

To investigate the relationship between HRQoL, SE and sociodemographic variables, Pearson’s product moment correlation coefficients were calculated. As Table V shows, the correlations between HRQoL and SE were negative and significant (*r*=−0.36, *p*<0.001). Of the sociodemographic variables, increasing age, not living with parents and number of moves had significant negative effects. Age correlated significantly with an increase in SE, longer residence time and not living with parents.

**Table V.** Pearson’s correlation between HRQoL Index, sociodemographic variables and stressful events.

Variables	1	2	3	4	5	6	7	8	9	10
1. HRQoL Index	–									
2. Age	−0.29***	-–								
3. Sex	0.03	0.32***	–							
4. Living with parents	0.27***	−0.47***	−0.29***	–						
5. Number of siblings	0.05	−0.28***	−0.30***	0.42***	–					
6. Residence time	−0.12	0.28***	0.13	−0.26	0.14	–				
7. Number of moves	−0.22**	0.16	0.17	−0.22**	−0.10	0.11	–			
8. Father’s education	**0.**04	0.10	0.06	−0.04	0.03	0.05	−0.05	–		
9. Mother’s education	0.12	0.02	−0.08	−0.06	0.04	−0.01	0.09	0.68***	–	
10. Missed schooling	0.01	0.01	0.24**	−0.01	0.15	−0.05	−0.14	0.12	0.09	
11. Stressful events	−0.36***	0.40***	0.26***	−0.33***	−0.31***	0.26**	0.27**	0.14	0.12	−0.12

Significance after Bonferroni correction shown in bold.

Correlation coefficient effect size (convention): 0–0.29=small; 0.30–0.49=moderate; 0.50–1.0=large.

Sex: 0= female; 1=male. Living with parents: 0=no; 1=yes.

* *p*⩽0.05; **p⩽0.01; ***p<0.001.

Hierarchal multiple regression analyses were done to examine the predictive value of SE and settlement variables on the HRQoL Index and the highest and lowest scoring dimensions (school, autonomy/parents and psychological well-being), controlling for age and sex (Table VI). The variables included were chosen based on the correlation analysis in Table V and previous research suggesting age, sex, family situation, residence time and war-related events are important determinants of HRQoL in immigrant youth [18,20]. The variables were added in chronology of occurrence, as suggested by other authors [6]. Age and sex were entered in step 1, explaining 10% of the variance in the HRQoL Index scores. After entering SE in step 2, the total variance explained was 18% (*F*(3, 127)=9.39, *p*<0.001). SE therefore explained an additional 8% of the variance in HRQoL Index scores after controlling for age and sex (*R*^2^ change=0.08, *F* change (1, 127)=12.93, *p*<0.001). After adding the settlement factors, the *p*-levels were similar in models 3 and 2, and the total variance explained was 21%. Repeating the same procedure for the two highest scoring dimensions, we found similar influences, with 19% of variance explained in autonomy/parents, but less of the variance was explained for school environment (12%). When repeated for the dimension with the lowest score, psychological well-being, 23% of the total variance was explained. In the fully adjusted models, being female decreased the scores significantly for the HRQoL Index and psychological well-being scores, and in the latter, increasing age reduced the scores significantly. Having experienced SE was a significant negative factor across all dimensions analysed. The other variables were not significant.

**Table VI.** Summary of hierarchical regression analysis for stressful events (SE), family situation, number of moves in the past five years and residence time, predicting dimensions of HRQoL, after controlling for age and sex.

Predictor		HRQoL Index				Autonomy/parents				School environment				Psychological well-being			
		*b*	SE *b*	β	*p*	*b*	SE *b*	β	*p*	*b*	SE *b*	β	*p*	*b*	SE *b*	β	*p*
Step 1																	
	Constant	116.05	14.20			118.50	17.10			97.24	17.31			121.03	16.60		
	Age	−2.50	0.67	−0.33	<0.001***	−2.50	0.80	−0.28	0.002**	−1.78	0.82	−0.20	0.032*	−3.05	0.78	−0.35	<0.001***
	Sex	5.03	3.28	0.14	0.127	2.90	3.95	0.07	0.465	−1.51	4.02	−0.03	0.709	4.98	3.83	0.12	0.196
Step 2																	
	Constant	112.17	13.63			114.30	16.57			93.32	17.00			116.29	15.84		
	Age	−1.66	0.68	−0.22	0.016*	−1.59	0.82	−0.18	0.056	−0.93	0.85	−0.10	0.275	−2.03	0.79	−0.23	0.011*
	Sex	6.83	3.20	0.18	0.035*	4.84	3.86	0.11	0.212	0.31	3.96	0.01	0.937	7.18	3.69	0.17	0.054
	SE	−2.08	0.58	−0.32	<0.001***	−2.26	0.70	−0.29	0.002**	−2.12	0.72	−0.27	0.004**	−2.55	0.67	−0.33	<0.001***
Step 3																	
	Constant	115.79	13.77			118.14	16.63			95.58	17.32			117.96	16.15		
	Age	−1.27	0.74	−0.17	0.087	−0.83	0.89	−0.09	0.352	−1.01	0.92	−0.11	0.278	−1.90	0.86	−0.22	0.029*
	Sex	7.94	3.22	0.21	0.015*	6.33	3.88	0.14	0.106	0.21	4.04	0.01	0.958	7.66	3.77	0.18	0.044*
	SE	−1.79	0.60	−0.27	0.003**	−1.76	0.72	−0.23	**0.016** *	−1.99	0.75	−0.26	0.009**	−2.44	0.70	−0.32	<0.001***
	Residence time	0.25	1.35	0.02	0.850	−1.64	1.63	−0.09	0.314	−0.02	1.69	−0.00	0.999	0.51	1.58	0.03	0.749
	Moves	−8.02	5.26	−0.13	0.130	−8.09	6.35	−0.11	0.205	−6.31	6.62	−0.09	0.342	−3.71	6.17	−0.05	0.548
	Living w/parents	5.78	4.05	0.13	0.156	8.49	4.90	0.17	0.086	−1.62	5.10	−0.03	0.751	2.46	4.76	0.05	0.606

Sex: 0= female; 1=male. Living with parents: 0=no; 1=yes.

* *p*⩽0.05; **p⩽0.01; ***p⩽0.001.

Index: *R*^2^=0.10*** for step 1, *∆R*^2^=0.08*** for step 2, *∆R*^2^=0.03 for step 3 (NS).

Autonomy and parents: *R*^2^=0.07** for step 1, *∆R*^2^=0.07** for step 2, *∆R*^2^=0.05 for step 3 (NS).

School environment: *R*^2^=0.05 (NS) for step 1, *∆R*^2^=0.06** for step 2, *∆R*^2^=0.01 for step 3 (NS).

Psychological well-being: *R*^2^=0.11*** for step 1, *∆R*^2^=0.09*** for step 2, *∆R*^2^=0.01 for step 3 (NS).

A sensitivity analysis including only the age group 13-18 years (n=95) showed that the effect of age as a predictor for psychological wellbeing was reduced (β -.22 vs -.08). This suggests that the effect of age is only present in the older age group (see Supplemental Material).

## Discussion

The main objectives of this study were to explore HRQoL in young Syrian refugees within the resettlement context in relation to norms from other youth populations, and to investigate if earlier SE and settlement factors contributed to their levels of HRQoL. We found that the level of HRQoL in the group was moderately good, but lower than population norms. Areas of concern were the dimensions of friends, physical well-being and psychological well-being, the latter showing the most difference from population norms. Areas of strength were school environment and autonomy/parents. Being female affected the scores negatively in terms of the HRQoL Index and psychological well-being, also with increasing age in the latter. These effects disappeared when young adults (19–24 years) were excluded. SE had a small and negative impact on scores in all dimensions. Settlement factors (residence time, number of times moved or living with parents) showed no influence, apart from living with parents which was significant only for the 13- to 18-year-olds in the dimension of autonomy/parents. The results will now be discussed in relation to each dimension, and the cultural and developmental sensitivity of the measurements.

### HRQoL Index

The overall HRQoL Index in our study population of young Syrians was moderately good, with an average of 64 for the age range 13–24 years and 67.5 for the age range 13–18 years. This is lower than European population norm data and Norwegian youth, with HRQoL Index scores of 72 [16,26]. The youngest age group in our study, 13- to 16-year-olds, had a HRQoL Index score of 71, similar to the population norm. Fewer studies have looked at HRQoL in refugee youth in particular, but a UN project assessed the HRQoL of Syrian children (median age 14 years) living in Jordan [21]. Their HRQoL Index score was 63, which is close to our result for the whole study population but much lower than the similar age group of 13- to 16-year-olds in our study, suggesting that not only age but also contextual factors contribute to the scores. The majority of our participants reported experiencing SE, which corresponds closely to other studies [4–6]. Boys and the oldest age groups reported more SE, possibly being less shielded from war events before leaving Syria. Combined with less support from family, as they were more likely to live alone, this might partly explain the lower HRQoL in the eldest age group in our sample.

### Physical well-being

This dimension had the lowest score in this study and was significantly lower than the population norm data. Some studies have found that exposure to violence is an important predictor for physical health [27], but this was not apparent in our study. Other studies have found high levels of somatic complaints in refugee youth, which might affect the scores in this dimension [28]. Self-rated health is an important predictor for future health [29], and the low scores indicate the need for interventions.

### Psychological well-being

This dimension explored positive and negative emotions, life satisfaction and self-esteem. A substantial portion of the literature concerning refugee youth shows a high prevalence of mental and emotional health problems [7]. These results correspond with the significantly low scores in the psychological well-being dimension in this study. Mental-health problems are strongly related to poorer HRQoL, although the two constructs are not synonymous [30]. Direct experience of adverse events is associated with an increased likelihood of psychological disturbance in refugee children and youth [7], and in our study SE explained 9% of the variance in psychological well-being after controlling for age and sex, suggesting that factors such as mental and emotional health problems are mediators to experienced HRQoL. The results indicate that psychological well-being is an area of concern for the whole group, not only for the youth with many experienced SE.^7^

### Autonomy and parent relations

This dimension explored the perceived autonomy, financial resources, interaction and support from parents. Lower scores for the oldest age group living alone could potentially be connected to financial resources. Family and home life is important for general well-being, and unaccompanied refugee minors are therefore regarded as an especially vulnerable group lacking the support from parents or family [7]. However, parental presence does not ensure protection. For example, parents are more likely than their children to have experienced violence from war, and their experiences are a stronger predictor than direct experiences of the children’s mental health [4,7]. Parental struggles are also associated with harsher parenting styles and higher levels of conduct problems in the children [4]. Adult Syrian refugees have high rates of SE and psychological symptoms [31], and we therefore expected that the satisfaction scores in this dimension would be low. Instead, it is one of the highest scored dimensions, similar to the European population norms, contributing strongly to positive HRQoL. Illustrating the complexity and importance of the family dimension, Daud et al. [32] found that the level of parental symptoms and family communication affected how the children developed either symptoms similar to their parents or forms of resilience. Addressing distress and supporting the families of refugee youth that live at home is therefore important to ensure that parental influence continues to be positive [10]. A holistic and family-oriented strategy is also seen as important in a recent guidance document from the World Health Organization (WHO), addressing the health needs of refugee and migrant children [33]. For those youth not living with parents or family, other support networks would be equally important to explore.

### Social support and peers

This dimension relates to both social support and friendships, vital to the experience of well-being for adolescents [34]. A strong peer attachment is also associated with greater levels of well-being in refugee youth [19]. Friends is the highest scoring dimension in the European population norm data, but in this study, the mean score was significantly lower. In many instances, friendship and social relationships are intertwined with the experience of belonging and being a valued member of a group or larger society, which is a central concept to integration [19]. The lower scores in this dimension, combined with rates of bullying and discrimination that are higher in migrant groups [11,20], strongly suggest that interventions need to focus on these factors.

### School environment

This dimension explores perceptions of concentration, learning, feelings about school and relationships with teachers. Many Syrian youth have interrupted and incomplete education before arrival [35], and our participants reported a mean of 4.4 years of missed schooling. They also need to learn a new language when they start school in Norway. School environment is the dimension with the lowest satisfaction in the European population norms, and minority youth are less satisfied with the school environment than majority youth [36]. We therefore expected mean scores in this dimension to be low, but instead it was the HRQoL dimension with one of the highest levels of satisfaction, with scores even higher than the norm for the younger participants. Of the variables included, only SE had a significant albeit small effect. Most of the participants attended introductory classes with a multi-ethnic make-up located in adult learning centres or upper secondary schools. These educational services might be more tailored to the needs of the group, and have staff with more knowledge of acculturation processes, contributing to higher student satisfaction. Another explanation might lie in the multi-ethnic make-up of the classes, as a Swedish study found a systematic tendency for students in schools with a high proportion of migrant youth, irrespective of their origin, to report higher levels of well-being and lower levels of bullying compared to less multi-ethnic schools [20]. The results reiterates schools’ important role not only as educational facilities but also as arenas for socialisation, integration and rehabilitation [37]. School-based programmes focussing on health and well-being therefore carry great potential, as is suggested by the recent guidance document from the WHO [33].

### Cultural, contextual and developmental sensitivity

When interpreting the present findings, the cultural and developmental sensitivity of the measurements needs to be considered. Cultural differences in perceptions of health, illness and the meaning of quality of life might influence responses. KIDSCREEN was therefore chosen due to its cross-cultural development and validation on several continents in order to ensure that concepts were culturally relevant with some level of universality [38]. Some studies indicate that migrants from Middle Eastern countries report lower levels of HRQoL than, for example, their African counterparts [18,20]. However, a sample of healthy youth in Jordan [39] reported HRQoL scores much closer to the European population norm data than Syrian refugees in Jordan [21] or our participants, suggesting that the context of being an immigrant or refugee might have influenced the scores rather than cultural interpretations. This is also illustrated by the small but persistent influence of experienced SE across all dimensions also found in other studies [18]. However, the relatively static sociodemographic and settlement factors included – residence time, number of moves and living with parents – did not correlate or affect HRQoL scores, which is also similar to other studies [18]. We found that length of residence time, a factor associated with reduced mental-health problems in refugees [13], did not affect the HRQoL scores in this study. This might be due to recruiting recent settlers, and that changes in HRQoL occur over longer time spans. Further investigations, possibly including more modifiable factors such as social support, acculturation and settlement stress over longer periods of time, might offer more insight into risks and resilience. Mental-health problems should also be explored further, as a higher prevalence is reported in refugees [4–10], which might reflect the lower scores in the psychological well-being dimension in this study [30].

The same consideration when interpreting the results should apply to developmental aspects, as developmental differences in cognitive, emotional, social and relational dimensions may affect the measure of HRQoL. In studies including children and adolescents, increasing age predicts a reduction in HRQoL [22,26], which is similar to our population. However, in this study, increasing age also increased the difference between our study sample and the population norms, suggesting a larger reduction in HRQoL than expected. Lower levels of HRQoL, often associated with higher levels of mental-health problems, have been reported in older adolescents [16]. This might account for the age differences seen in the HRQoL Index and psychological well-being scores, which disappeared in the sensitivity analysis.

For some of the HRQoL dimensions in this study, the developmental context varied greatly between a 13-year-old and a 24-year-old, especially for autonomy and parental relations. Other studies using KIDSCREEN-27 in young adults also suggest that autonomy/parents must be interpreted with more caution for older age groups. However, a Rasch evaluation also suggests acceptable results for all other dimensions in the KIDSCREEN-27 [24].

### Strengths and limitations

One important shortcoming is the lack of information on the actual number of Syrian students attending the schools included in the study, as students’ country of origin is not normally noted in their records. The total attrition rate and whether participants and non-participants differed in any respect is not known, and evaluating their representativeness is difficult. There were fewer girls than boys participating. However, this reflects the sex distribution for the age group residing in Norway. Although care was taken to inform the participants, language difficulties, fear or compliance might have affected their answers and might have contributed to the difficulties in obtaining consent forms from parents. Considering the high levels of poor mental health in unaccompanied refugee youth [7], it would have been beneficial to have information about their immigration status or contact with their family, especially for those registering as ‘living alone’. Lastly, quality of life is not static. Therefore, longitudinal or prospective studies would provide changes over time. Due to its cross-sectional design, it is not possible to make causal inferences from the study.

The strengths include the use of well-validated measures and recruiting resettled refugee youth living with their families. Using HRQoL highlights its importance as an important outcome that enables an ecological and developmental perspective, describing the health and well-being of a whole population to complement other measurements. A common critique of studies on migration and health is the heterogeneity of participants originating from all over the world [7], which this study avoids to some extent. However, this limited the size of the sample, making it harder to generalise findings.

## Conclusions

For most participants, the overall HRQoL was moderately good, despite possible risk factors. Autonomy/parents and school environment contributed the most to a positive HRQoL. Friends, physical well-being and especially psychological well-being were lower than population norms, highlighting areas of strength and concern. Sociodemographic factors had little explanatory value, but SE had an impact across all dimensions. Age was only predictive for reduced psychological wellbeing in the eldest age group. Total variance explained was only 21%, suggesting that more modifiable factors are relevant to explore in future research.

### Implications

For future research, HRQoL is a non-invasive measure with the potential of exploring dimensions of strengths and concerns, and an important complement to mental-health measures in studies of refugees. To improve contextual relevance, the generic HRQoL measurements could be augmented by adapting the general measure or developing more migration or refugee specific modules. These modules could include impact or stress related to acculturation, identity or discrimination, for example, inspired by how disease-specific modules in HRQoL measures for disabled or chronically ill children have been developed (DISABKIDS [40]).

For schools, this study illustrates their importance as a resource for well-being, and this role needs to be continually supported. Teachers’ competence and multicultural environments might be important contributing factors, since lower school satisfaction is reported in other studies. Being a safe arena means interventions targeting areas of concern, such as social support and psychological well-being, could be based here. Such interventions could be network building, mentorships and school-based mental-health services.

For public-health professionals, this study shows that HRQoL was good for most youth, despite difficult circumstances, suggesting resilience. Parents seemed to be an important resource, emphasising the importance of including families in interventions. SE showed a small but consistent explanatory power, indicating that traumatic events are central. However, more universal public-health interventions targeting a wider range of mental-health problems might be just as beneficial, especially as HRQoL might also be a valuable resource for negotiating other settlement stressors in refugee youth.

## Supplemental Material

SJP929833_Supplemental_material – Supplemental material for Health-related quality of life in young Syrian refugees recently resettled in Norway Click here for additional data file. Supplemental material, SJP929833_Supplemental_material for Health-related quality of life in young Syrian refugees recently resettled in Norway by Cecilie R. Dangmann, Øivind Solberg, Anne K.M. Steffenak, Sevald Høye and Per N. Andersen in Scandinavian Journal of Public Health
