# Supplementary material for: Health-related quality of life in young Syrian refugees recently resettled in Norway
Source: Scand J Public Health. 2020 Jul 2;48(7):688–98. doi: 10.1177/1403494820929833 (PMC7604933; doi:10.1177/1403494820929833)
Supplement: SJP929833_Supplemental_material – Supplemental material for Health-related quality of life in young Syrian refugees recently resettled in Norway [file SJP929833_Supplemental_material.pdf]

Table 7. Sensitivity analysis for age including only 13-18 year olds (n=5): Hierarchical regression analysis for stressful events (SE), family situation, number of moves in the past five years and residence time, predicting dimensions of HRQoL, after controlling for age and gender.

Predictor		HRQoL Index (n=93)				Autonomy/Parents (n=92)				School environment (n=95)				Psychological wellbeing (n=94)			
		b	SE b	β	*p*	b	SE b	β	*p*	b	SE b	β	*p*	b	SE b	β	*p*
Step 1																	
	Constant	114.84	29.57			119.17	35.330			100.05	35.517			129.95	34.13		
	Age	-2.63	1.73	-.18	.131	-2.50	2.06	-.14	.230	-2.36	2.07	-.13	.259	-3.75	1.99	-.21	.064
	Sex	-2.80	4.21	-.08	.507	-3.46	5.03	-.08	.494	6.09	5.06	.14	.233	3.68	4.86	.09	0.451
Step 2																	
	Constant	96.41	28.76			98.81	34.66			80.42	34.99			107.09	32.87		
	Age	-.81	1.75	-.05	.645	-.48	2.11	-.03	.820	-.42	2.1	-.02	.845	-1.49	2.00	-.09	.460
	Sex	-4.78	4.05	-.13	.242	-5.65	4.89	-.13	.252	3.98	4.93	.09	.423	6.14	4.63	.14	.189
	SE	-2.40	.80	-.35	**.004****	-2.65	.97	-.33	**.008***	-2.56	.98	-.31	**.011****	-2.98	.92	-.38	**<.002 *****
Step 3																	
	Constant	113.31	29.87			122.23	35.55			79.24	37.35			113.07	34.92		
	Age	-.87	1.75	-.06	.623	-.34	2.09	-.02	.873	-.32	2.19	-.02	.884	-1.33	2.05	-.08	.519
	Sex	-6.98	4.17	-.19	.098	-8.57	4.96	-.20	.089	4.15	5.21	.09	.429	6.84	4.88	.16	.165
	SE	-1.81	.86	-.27	.040*	-1.81	1.03	-.22	.082	-2.60	1.08	-.32	.019	-2.76	1.01	-.35	**.008****
	Residence time	.01	1.89	.001	.995	-.64	2.25	-.03	.777	-.15	2.36	-.01	.949	-.43	2.21	-.02	.847
	Moves	-9.94	7.89	-.15	.212	-7.45	9.39	-.10	.430	3.14	9.87	.04	.751	1.38	9.23	.02	.881
	Living w/parents	-9.44	7.48	-.15	.211	-18.20	8.90	-.25	.045	-1.52	9.35	-.02	.872	-7.45	8.75	-.10	.397

Only 13-18 year olds included in the analysis.

Sex: 0= female 1=male. Living with parents 0=No 1 =Yes.
**p* ≤.05; ***p* ≤0.01 ****p* ≤0.001.
HRQoL Index: R2 = .03 (NS) for step 1, ΔR2= .11** for step 2, ΔR2= .05 for step 3 (NS) (Total model R^2= .188, Adjusted R^2= .118)
Autonomy and Parents: R^2 = .02 (NS) for step 1, ΔR^2= .09** for step 2, ΔR^2= .07 for step 3 (NS) (Total model R^2= .185, Adjusted R^2= .115)
School environment: R^2 = .04 (NS) for step 1, ΔR^2= .08** for step 2, ΔR^2= .01 for step 3 (NS) (Total model R^2= .123, Adjusted R^2= .048)
Psychological wellbeing: R^2 = .05 (NS) for step 1, ΔR^2= .12*** for step 2, ΔR^2= .01 for step 3 (NS) (Total model R^2= .180, Adjusted R^2= .110)
